# Frontal Structural Neural Correlates of Working Memory Performance in Older Adults

**DOI:** 10.3389/fnagi.2016.00328

**Published:** 2017-01-04

**Authors:** Nicole R. Nissim, Andrew M. O’Shea, Vaughn Bryant, Eric C. Porges, Ronald Cohen, Adam J. Woods

**Affiliations:** ^1^Center for Cognitive Aging and Memory, McKnight Brain Institute, Department of Clinical and Health Psychology, University of FloridaGainesville, FL, USA; ^2^Department of Neuroscience, University of FloridaGainesville, FL, USA

**Keywords:** cognitive aging, cortical surface area, cortical thickness, FreeSurfer, N-Back, Structural Magnetic Resonance Imaging

## Abstract

Working memory is an executive memory process that allows transitional information to be held and manipulated temporarily in memory stores before being forgotten or encoded into long-term memory. Working memory is necessary for everyday decision-making and problem solving, making it a fundamental process in the daily lives of older adults. Working memory relies heavily on frontal lobe structures and is known to decline with age. The current study aimed to determine the neural correlates of decreased working memory performance in the frontal lobes by comparing cortical thickness and cortical surface area from two demographically matched groups of healthy older adults, free from cognitive impairment, with high versus low N-Back working memory performance (*N* = 56; average age = 70.29 ± 10.64). High-resolution structural T1-weighted images (1 mm isotropic voxels) were obtained on a 3T Philips MRI scanner. When compared to high performers, low performers exhibited significantly decreased cortical surface area in three frontal lobe regions lateralized to the right hemisphere: medial orbital frontal gyrus, inferior frontal gyrus, and superior frontal gyrus (FDR *p* < 0.05). There were no significant differences in cortical thickness between groups, a proxy for neurodegenerative tissue loss. Our results suggest that decreases in cortical surface area (a proxy for brain structural integrity) in right frontal regions may underlie age-related decline of working memory function.

## Introduction

Working memory is a vital process underlying human thought. Working memory is a limited capacity system that involves active manipulation of information currently being maintained in focal attention (Glisky, 2007). Working memory is one component of executive function that allows for transitional information to be held and manipulated temporarily in memory stores, before either being forgotten or encoded into long-term memory (Baddeley, 1992; Goldman-Rakic, 1996; Owen et al., 2005). As with other components of executive function, working memory processes rely heavily on frontal lobe structures (Courtney et al., 1998). Working memory processes guide voluntary or goal-directed behaviors including short-term maintenance of relevant information, mental manipulations, and mental organization of imminent sequence of actions (Goldman-Rakic, 1987; Boisgueheneuc et al., 2006). Working memory is necessary for everyday decision-making and problem-solving, making it a fundamental process in the lives of older adults. Activities of daily living such as preparing meals, taking medication, paying bills, as well as organizing and planning daily routines and appointments require working memory and other components of executive function (Mograbi et al., 2014). As such, declines in working memory can lead to deficits in these domains and consequently lead to loss of independence and decreased quality of life (Klingberg, 2010; Williams and Kemper, 2010). Working memory performance can be impacted by age-related reductions in working memory capacity and is increasingly susceptible to interference in older adults. Not surprisingly, memory loss and perceived declines in memory performance are frequent complaints in older adult populations (Gazzaley et al., 2007; Kaup et al., 2014). As frontal cortices undergo the most pronounced structural decline with advanced age (Lemaitre et al., 2012) and play an important role in working memory function (Freeman et al., 2008), identifying frontal structures underlying age-related working memory decline may provide important therapeutic targets for combating cognitive aging.

The prefrontal cortex participates in cognitive features of behavior, engaging the organization of goal-directed behaviors (Fuster, 1988). Although the frontal lobe is the last brain region to mature in humans around age 25, it is also one of the first regions to structurally decline during the aging process, following the ‘last in, first out’ model of aging (Salat et al., 2004). Studies of brain morphometry show that the prefrontal cortex experiences the most striking reductions (Lemaitre et al., 2012). Similarly, age-related decreases in cortical surface area are greatest in frontal regions (Salat et al., 2004), while the greatest age-related volume reductions occur in the middle frontal gyrus, the superior frontal gyrus (SFG), and the frontal pole (Lemaitre et al., 2012).

The frontal lobes, and the right frontal lobe in particular, play an important role in working memory function. The ability to hold onto visuospatial information, to be fractioned into separate visual and spatial components, is thought to be principally represented within the right hemisphere (Baddeley, 2000). Prabhakaran et al. (2000) compared the retention of verbal and spatial information held in integrated or unintegrated forms using functional magnetic resonance imaging (fMRI), and found greater right frontal activation for integrated information, providing evidence for the right frontal lobe being particularly critical for retention of integrated information (Baddeley, 2000; Prabhakaran et al., 2000). Previous fMRI work studying the functional neural basis of aging and working memory have shown distinct activation patterns in older versus younger adults, and for high versus low performance rates on an N-Back working memory task (Cabeza, 2002; Dolcos et al., 2002). Positron emission tomography (PET) and fMRI studies of higher-order cognitive functions have been associated with prominent activations in the prefrontal cortex. Often, activations are sometimes lateralized, which may reflect the nature of the processes and/or the stimuli involved (Nyberg et al., 1996; Cabeza et al., 2002). Prefrontal cortex activity tends to be less asymmetrical in older than younger adults (Cabeza et al., 2002). Young high performers on working memory tasks tend to exhibit significant activation of the dorsolateral prefrontal cortex (DLPFC) lateralized to the right hemisphere. Older adults with low performance exhibit more robust right hemisphere activation than young, potentially reflecting inefficiency of activation, whereas older adults who perform at the same level as young adults exhibit bilateral activation patterns in the prefrontal cortex. This difference in activation patterns of high performing older adults compared to high performing younger adults may counteract age-related neurocognitive declines as a form of compensatory mechanism (compensation hypothesis), or it could reflect age-related deficits in recruiting specialized neural mechanisms (dedifferentiation hypothesis; Cabeza et al., 2002). While the functional pattern of working memory performance in older adults has been well explored, the age-related structural alterations in frontal cortices underlying working memory decline versus compensation remains unclear.

Older adults exhibit significant deficits in tasks that involve active manipulation, reorganization, or integration of the contents of working memory (Salthouse et al., 1989). Investigating the structural neural correlates of performance on working memory tasks in older adults is necessary to understand how working memory systems change with age. This study aimed to determine the frontal structures underlying poorer working memory performance. We hypothesized that working memory deficits would be associated with decreases in cortical surface area in right frontal brain regions in healthy older adults. We did not expect any significant changes in cortical thickness, as decreases in thickness signify neurodegenerative tissue loss (Shefer, 1973; Fischl and Dale, 2000) and our population of interest was healthy older adults. In contrast, cortical surface area serves as a proxy for gray matter structural integrity (Fischl and Dale, 2000; Salat et al., 2004; Dickstein et al., 2007; Lemaitre et al., 2012).

## Materials and Methods

### Participants

We recruited healthy community dwelling older individuals in the Gainesville and North Florida region (*N* = 56, 50% female, 52 right handers). A thorough medical history questionnaire for each participant provided detailed information on health status, medication status, and allowed us to rule out the presence of age-related brain disorders. Exclusionary criteria for the study included pre-existing neurological or psychiatric brain disorders, MRI exclusions, mild cognitive impairment (MCI) or diagnosis with a neurodegenerative brain disease (i.e., dementia or Alzheimer’s). The Montreal Cognitive Assessment (MoCA) was given to assess general cognitive ability and rule out possible MCI (Nasreddine et al., 2005). Additionally, the MoCA allowed us to control for differences in global cognitive function and insure our analyses were directly relevant to working memory rather than a reflection of generalized cognitive deficits. The MoCA cut off score to be an eligible participant in the study was 20. A comprehensive neuropsychological battery was performed on each participant to provide for clinical assessment of MCI status. A clinical neuropsychologist assessed participant performance on the battery to determine MCI status. No participants in this sample were clinically indicated to have MCI using this approach. Participants did not significantly differ in age, sex, education, MoCA score, or intracranial volume (ICV; *p* > 0.05). ICV is especially important to control for as it is closely relates to brain size (Hentschel and Kruggel, 2004; Im et al., 2008), and thus was also included as a covariate in our model to rule out the possibility of head size driving any cortical thickness or cortical surface area differences between groups. The total sample (*N* = 56) consisted of 28 female and 28 male older adults. The range for the total sample of the following covariates were: MoCA scores = 20–30, age = 44–89 years old, education = 12–20 years, and ICV = 975547.27–1988968.30. See **Table 1** for demographic means, standard deviations, and statistics for the total sample. All participants in the study underwent cognitive testing followed by an MRI scanning session where the N-Back task was performed inside the scanner. fMRI data on N-Back will be presented in a subsequent paper. N-Back performance data was used to characterize participants into high and low working memory groups (described in detail below). Prior to any study procedures, all participants provided written informed consent. The study protocol was carried out in accordance with the Declaration of Helsinki, and the University of Florida Institutional Review Board approved all procedures in this study.

**Table 1:** Demographic data and independent *t*-test statistics (means, standard deviation).

	Total sample	High performers	Low performers	Independent *t*-test
	*N* = 56	*n* = 29	*n* = 27	*t* (*p*)
Age	70.29 (10.64)	68.00 (11.06)	72.74 (9.77)	-1.695 (0.096)
Sex	28F:28M	14F:15M	14F:13M	-0.263 (0.794)
Education	16.30 (2.33)	16.24 (1.88)	16.37 (2.76)	-0.203 (0.840)
MoCA total score	25.89 (2.67)	26.48 (2.69)	25.26 (2.55)	1.742 (0.087)
Intracranial volume	1443534.46 (225849.81)	1457084.28 (216839.60)	1428980.96 (238413.09)	0.462 (0.646)

### N-Back Task

**FIGURE 1:**
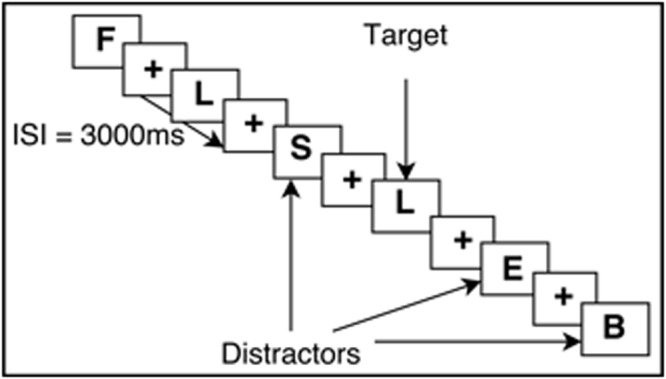
**Example of the 2-Back working memory paradigm**. ISI, Interstimulus interval.

The N-Back task requires continuous performance in which participants are asked to monitor the identity of a series of stimuli and indicate when the currently presented stimulus is the same as the one presented *n*-trials previously (Kirchner, 1958; Owen et al., 2005). This task is known to engage working memory processes and thus was used in this study. Participants completed an N-Back practice session to ensure that all instructions were clear and that participants could accurately perform the task. All N-Back tasks were created with E-Prime version 2.0 (Psychology Software Tools Inc., Pittsburgh, PA, USA). The task was completed inside the scanner, with images projected onto a screen behind the participants’ head and viewed through a mirror mounted on the head coil. Responses were made via an MRI-compatible button box, using the middle and index finger. Participants performed two runs of the N-Back, which included both 0-Back and a 2-Back version of the N-Back, totaling 15 min of functional task time. For 0-Back, participants were asked to respond by button press (with index finger) when they saw a X on the screen, and respond with another button press (with middle finger) when they saw any other letter (distractors). This task was used as an attention control. Each letter was displayed one at a time, for 700 ms, followed by a crosshair for 2300 ms. The participants could respond by button press at any point in the total 3000 ms trial interval. During the 2-Back task, participants viewed single letters (i.e., letters of identical font, color, size) on the screen with the same timing scheme as 0-Back. When a letter appeared and was the same as the letter that was presented two letters prior, participants were asked to respond to that target letter by a button press of their index finger (see **Figure 1** for visual example). All letters that did not match the 2-Back pattern were used as distractors, and participants were asked to respond by button press with their middle finger. The order of whether participants received 2-Back or 0-Back first was randomized.

### N-Back Working Memory Performance Characterization

N-Back accuracy rates were collected and recorded in E-Prime v2.0 then transferred as total percent accuracy scores of both runs into SPSS. The data was then processed through SPSS version 21. All participants responded to greater than 75% of all N-back trials. A median split based on 2-Back accuracy was performed to determine high versus low performers. High performers (*N* = 29) scored 67% or above correctly on 2-Back, while low performers (*N* = 27) had an accuracy score of 66% or below. For 5 participants (three high performers, two low performers), one of their runs was lost during data collection due to technical problems. For these participants, the one run collected was used for analyses.

### Working Memory Group Demographics

Behavioral data for 0-Back average accuracy was 83.71 ± 17.38% (range = 19–98%) while 2-Back average accuracy was 64.88 ± 16.93% (range = 20–90%) for the overall sample (*N* = 56). High and low working memory performers on the 2-Back task were determined by performing a median split of accuracy scores. Accuracy scores of 67% or above were grouped as high performers. In contrast, scores below 67%, were grouped as low performers. See **Table 2** for more detailed task performance information for high and low groups. The range for the following covariates for the low performing group was: age = 47–89, education = 12–20, MoCA = 20–30, ICV = 1001786.49–1954571.14. The range for the following covariates for the high performing group was: age = 44–85, education = 12–20, MoCA = 21–30, ICV = 975547.27–1988968.30. High versus low working memory groups did not significantly differ on the above covariates (*p* > 0.05). For high and low group demographic means, standard deviations, and test statistics, see **Table 1**.

**Table 2:** Average N-Back accuracy scores and standard deviation.

	High performers	Low performers	Total sample
	*n* = 29	*n* = 27	*N* = 56
2-Back	77.90% (7.04)	50.89% (12.69)	64.87% (16.93)
0-Back	90.79% (8.82)	76.11% (21.75)	83.71% (17.83)

### MRI Acquisition

T1-weighted MPRAGE structural MRI scans were performed on all participants. Participants were imaged in a Philips Achieva 3.0 Tesla (3T) scanner (Philips Electronics, Amsterdam, The Netherlands) with a 32-channel receive-only head coil. Scan parameters: repetition time (TR) = 7.0 ms; echo time (TE) = 3.2 ms; flip angle = 8°; field of view = 240 mm × 240 mm × 170 mm; voxel = 1 mm × 1 mm × 1 mm. Foam padding was placed around the head to limit motion during the scan. No images exhibited evidence of motion artifact. Participants were given headphones and earplugs to minimize noise while inside the scanner.

### T_1_-Weighted Neuroimaging Processing

Cortical reconstruction and volumetric segmentation was performed with FreeSurfer version 5.3 image analysis suite. The technical details of these procedures are described in prior publications (Dale and Sereno, 1993; Dale et al., 1999; Fischl et al., 1999a,b, 2001, 2002, 2004a,b; Fischl and Dale, 2000; Segonne et al., 2004; Han et al., 2006; Jovicich et al., 2006). Briefly, this processing includes removal of non-brain tissue (Segonne et al., 2004), automated Talairach transformation, segmentation of the subcortical white matter and deep gray matter volumetric structures (Fischl et al., 2002, 2004a), intensity normalization (Sled et al., 1998), tessellation of the gray matter white matter boundary, automated topology correction (Fischl et al., 2001; Segonne et al., 2007), and surface deformation following intensity gradients to optimally place the gray/white and gray/cerebrospinal fluid borders at the location where the greatest shift in intensity defines the transition to the other tissue class (Dale and Sereno, 1993; Dale et al., 1999; Fischl and Dale, 2000). Once the cortical models are complete, a number of deformable procedures can be performed for in further data processing and analysis including surface inflation (Fischl et al., 1999a), registration to a spherical atlas which utilized individual cortical folding patterns to match cortical geometry across subjects (Fischl et al., 1999b), parcellation of the cerebral cortex into units based on gyral and sulcal structure (Fischl et al., 2004b; Desikan et al., 2006). This method uses both intensity and continuity information from the entire three dimensional volume in segmentation and deformation procedures to produce representations of cortical thickness, calculated as the closest distance from the gray/white boundary to the gray/CSF boundary at each vertex on the tessellated surface (Fischl and Dale, 2000). The maps are created using spatial intensity gradients across tissue classes and are therefore not simply reliant on absolute signal intensity. The maps produced are not restricted to the voxel resolution of the original data and thus are capable of detecting submillimeter differences between groups. FreeSurfer measures have been shown to be both reliable and valid. Procedures for the measurement of cortical thickness have been validated against histological analysis (Rosas et al., 2002) and manual measurements (Kuperberg et al., 2003; Salat et al., 2004). FreeSurfer morphometric procedures have been demonstrated to show good test–retest reliability across scanner manufacturers and across field strengths (Han et al., 2006; Reuter et al., 2012). Once processed through FreeSurfer, all output was visually inspected for processing errors (e.g., mislabeling white matter, gray matter, or skull inclusions) and manually corrected for when necessary.

### Neuroimaging Measures: Cortical Thickness and Cortical Surface Area

The relationship between cortical surface area and cortical thickness creates a quantifiable brain volume. For example, although two objects may have the exact same volume, the shape or contours of the objects can vary considerably, exhibiting very different topography. If we consider a cube measuring 3 × 3 × 3 versus a rectangular shape measuring 3 × 9 × 1, both shapes share the same volume of 27 mm^3^; this exemplifies that surface area and thickness may exhibit a very different pattern while sharing the same volume. When we consider the human brain, age-related changes in surface area versus thickness may have different implications for behavioral and cognitive processes. These two components exhibit distinct patterns of change when comparing healthy versus diseased brains (Dotson et al., 2015). Gray matter, which makes up the cortical ribbon, experiences volume loss throughout adulthood into advanced age (Scott and Thacker, 2004). Neuronal density is relatively stable throughout life; any robust decrease in neuronal density is thought to reflect a disease state (Morrison and Hof, 2002; Dickstein et al., 2007). Decrease in cortical thickness is a proxy for neuronal loss due to neurodegenerative disease (Shefer, 1973; Fischl and Dale, 2000). While changes in cortical surface area and its relationship to general cognitive function is less known (Schnack et al., 2015), cortical surface area is thought to reflect the structural integrity of gray matter (Fischl and Dale, 2000; Salat et al., 2004; Lemaitre et al., 2012). It has been suggested that preservation in neuronal number, but loss of neuronal dendritic architecture underlies neocortical volume loss with increasing age in the absence of Alzheimer’s disease (Morrison and Hof, 2002; Freeman et al., 2008). In normal healthy aging, to our best knowledge, there are no studies that have closely examined cortical surface area changes and the possible role this may play in driving age-related declines in working memory function.

### Regions of Interest and Neuroimaging Statistical Analyses

Frontal lobe regions (defined as all regions anterior to the pre-central gyrus using the Desikan-Killiany parcellation, see **Table 3** for a comprehensive list of ROIs) and two control regions outside the frontal loges (left and right pericalcarine areas of the occipital cortex; i.e., V1; Desikan et al., 2006) were analyzed for both thickness and area using separate univariate general linear models with performance group (high versus low) as a fixed factor and age, sex, years of education, ICV and MoCA score as covariates using the software SPSS version 21. Control sites were included to assess the regional specificity of our frontal focused analyses. To control for multiple comparison type I error we implemented a false discovery rate (Benjamini and Hochberg, 1995) threshold of FDR < 0.05 using the software R, which is freely available for download online^1^.

**Table 3:** Surface area and thickness measures from frontal ROIs and control brain region.

Brain region	*F*-value	*P*-value	$\eta_{p}^{2}$	P-FDR
**Measurement: surface area**				
R Medial orbital frontal gyrus	10.99	0.002	0.183	0.018^∗^


R Superior frontal gyrus	9.84	0.003	0.167	0.018^∗^


R Pars opercularis	8.211	0.006	0.144	0.024^∗^


L Rostral anterior cingulate	5.815	0.02	0.106	0.24


L Medial orbital frontal gyrus	2.553	0.117	0.05	0.448


R Pars orbitalis	2.198	0.145	0.043	0.435


L Pars orbitalis	1.831	0.182	0.036	0.448


L Superior frontal gyrus	1.669	0.202	0.033	0.448


L Frontal pole	1.308	0.258	0.026	0.448


L Rostral middle frontal gyrus	1.185	0.282	0.024	0.448


R Pars triangularis	1.118	0.295	0.022	0.708


L Caudal middle frontal gyrus	1.11	0.297	0.022	0.448


L Lateral orbital frontal gyrus	0.947	0.335	0.019	0.448


L Caudal anterior cingulate	0.943	0.336	0.019	0.448


R Frontal pole	0.784	0.38	0.016	0.76


L Pars triangularis	0.55	0.462	0.011	0.554


R Caudal anterior cingulate	0.542	0.472	0.011	0.786


L Pars opercularis	0.282	0.598	0.006	0.652


R Rostral middle frontal gyrus	0.245	0.623	0.005	0.786


R Lateral orbital frontal	0.196	0.66	0.004	0.786


R Caudal middle frontal	0.143	0.707	0.003	0.786


R Rostral anterior cingulate	0.129	0.721	0.003	0.786


L Pericalcarine (control region)	0.109	0.742	0.002	0.742
R Pericalcarine (control region)	0.007	0.935	0.000139	0.935


**Measurement: thickness**				


L Pars triangularis	6.075	0.017	0.11	0.204


R Medial orbital frontal gyrus	4.851	0.032	0.09	0.261


R Pars opercularis	3.312	0.075	0.063	0.261


L Rostral anterior cingulate	3.319	0.075	0.063	0.295


R Caudal anterior cingulate	3.291	0.076	0.063	0.261


R Pars orbitalis	3.055	0.087	0.059	0.261


L Medial orbital frontal gyrus	2.88	0.096	0.056	0.295
L Caudal anterior cingulate	2.642	0.11	0.051	0.295
L Frontal pole	2.458	0.123	0.048	0.295
R Superior frontal gyrus	2.165	0.148	0.042	0.355
L Pars orbitalis	1.801	0.186	0.035	0.361
R Rostral anterior cingulate	1.489	0.228	0.029	0.413
L Pericalcarine (control region)	1.453	0.234	0.029	0.361
R Pericalcarine (control region)	1.41	0.241	0.028	0.413
L Pars opercularis	1.302	0.259	0.026	0.361
L Lateral orbital frontal gyrus	1.238	0.271	0.025	0.361
R Rostral middle frontal gyrus	1.187	0.281	0.024	0.421
L Superior frontal gyrus	1.001	0.322	0.02	0.386
L Rostral middle frontal gyrus	0.722	0.4	0.015	0.436
R Frontal pole	0.687	0.411	0.014	0.538
R Pars triangularis	0.484	0.49	0.01	0.538
R Caudal middle frontal gyrus	0.474	0.494	0.01	0.538
L Caudal middle frontal gyrus	0.33	0.568	0.007	0.568
R Lateral orbital frontal gyrus	0.014	0.908	0.000278	0.908

*R, Right, L, Left, ^∗^ = FDR < 0.05*.

## Results

### Cortical Surface Area and Thickness

**FIGURE 2:**
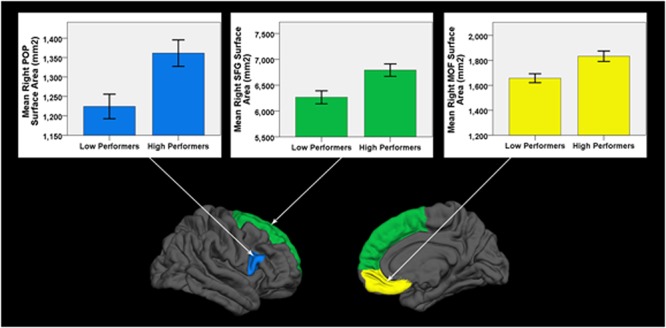
**Cortical surface differences between low versus high working memory performers**. Arrows connect graphs of between group differences to the affiliated gyri ROI highlighted on a FreeSurfer brain model. POP, pars opercularis of the inferior frontal gyrus; SFG, superior frontal gyrus; MOF, medial orbital frontal gyrus. Error bars = ± 1 SE.

Low performers exhibited significantly less surface area in three frontal lobe regions lateralized to the right hemisphere: SFG (*p*_FDR_ = 0.018; Cohen’s *D* = 0.81; surface area of high performers = 6791.59 ± 648.62 mm^2^; low performers = 6266.26 ± 649.15 mm^2^), pars opercularis of the inferior frontal gyrus (*p*_FDR_ = 0.024; Cohen’s *D* = 0.79; surface area of high performers = 1361.38 ± 183.34 mm^2^; low performers = 1224.19 ± 163.97 mm^2^), and medial orbital frontal gyrus [*p*_FDR_ = 0.018; Cohen’s *D* = 0.85; surface area of high performers = 1833.14 ± (225.09) mm^2^; low performers = 1657.00 ± 186.57 mm^2^]. No significant differences in cortical thickness were observed after correcting for multiple comparisons (FDR > 0.05). As a control brain region, the pericalcarine gyrus of the occipital lobe was analyzed in both hemispheres and did not significantly differ in thickness or surface area between groups. See **Figure 2** for significant surface area results and **Table 3** for all surface area and thickness results.

## Discussion

The current study investigated the neural correlates of age-related decreases in working memory performance in frontal cortices. We found significant differences in cortical surface area for three regions of the right frontal lobe. Low working memory performers had significantly less surface area for the inferior frontal gyrus (pars opercularis), SFG, and the medial orbital frontal gyrus, when compared to high working memory performers. These areas of decreased structural integrity are consistent with prior fMRI findings for functional correlates of working memory performance (Curtis and D’Esposito, 2003; Owen et al., 2005). These results are also consistent with prior research demonstrating right lateralized BOLD activation of frontal cortices in young adults with high working memory performance, but bilateral (potentially compensatory) activation of right and left frontal cortices in older adults able to maintain a high level of working memory performance (Cabeza et al., 2002). In contrast, older adults unable to maintain performance showed unilateral increase in activation of right frontal regions, perhaps consistent with less efficient neural processing (Cabeza et al., 2002). Collectively, our structural MRI findings, when considered in concert with prior functional MRI research (Cabeza et al., 2002), suggests that areas in the right prefrontal cortex are critical substrates for age-related change in working memory function. Our findings provide evidence that right lateralized structural abnormalities in inferior, superior, and medial orbital frontal gyri underlie age-related working memory decline.

### Pars Opercularis of the Inferior Frontal Gyrus

The pars opercularis (BA44), a sub-region of the inferior frontal gyrus, is included in the functionally defined ventrolateral prefrontal cortex (VLPFC; Molnar-Szakacs et al., 2005). The VLPFC is consistently found to be active in working memory fMRI studies; early functional neuroimaging studies that activated this region in humans tended to emphasize the explicit retrieval of one or a few pieces of information, as well as the sequencing of responses based directly on stored information (Owen et al., 2005). Aron et al. (2004) argues that the right VLPFC plays a critical role in cognitive inhibition. Cognitive inhibition is a component of executive control that can be localized to the right inferior frontal gyrus, specifically the pars opercularis (Molnar-Szakacs et al., 2005; Falquez et al., 2014). Inhibition can be defined as the suppression of inappropriate responses (Aron et al., 2004). Cognitive inhibition could be one of a set of functions (including working memory maintenance of task sets and items, selection and manipulation of information in working memory, and conflict detection) implemented by different, possibly overlapping prefrontal cortical regions. The voluntary blocking of memory retrieval may also be dependent on this region. As more information in the environment is perceived than can accurately and appropriately be attended to, inhibition is an integral feature of the prefrontal cortex that allows irrelevant information to be inhibited enabling more important information to be processed more quickly and efficiently.

### Superior Frontal Gyrus

The SFG is a large region of the prefrontal cortex, making up about 1/3 of the frontal lobe in the human brain. The SFG is thought to contribute to higher cognitive functions, and play a particularly important role in working memory (Boisgueheneuc et al., 2006). The functional anatomical region referred to as the DLPFC (BA9) overlaps structurally, in part, with SFG (Owen et al., 2005; Falquez et al., 2014).

The DLPFC plays a crucial role in terms of working memory. It has been established as a crucial node that supports working memory processes. Neurophysiological unit recordings of the DLPFC in monkeys have shown persistent sustained levels of neuronal firing during retention intervals of delayed response tasks (Curtis and D’Esposito, 2003). Sustained activity in the DLPFC is thought to provide a bridge between the stimulus cue and its contingent response (i.e., goal-directed behavior) in a working memory task. Goldman-Rakic (1987) has shown that lesions in the DLPFC impair the ability to maintain sensory representations on-line that are no longer present in the external environment. Studies of patients with SFG lesions show global impairments in working memory tasks with impairments present months to years post-lesion, indicating that the SFG may be a key component in the working memory network (Boisgueheneuc et al., 2006).

### Medial Orbital Frontal Cortex

The orbitofrontal cortex in primates is situated ventrally and frontally in the brain (Kringelbach and Rolls, 2004), and can be further divided into distinct areas. Two major subdivisions have been cytoarchitecturally and functionally identified: the lateral orbitofrontal cortex and medial orbitofrontal cortex (MOF). The medial orbital frontal cortex surface includes BA14 (Rolls, 2004).

The MOF receives input from all sensory modalities. Accumulating evidence from fMRI implicates the orbitofrontal cortex as a necessary component in working memory, demonstrating activity in this area while coordinating multiple working memory operations (Wager and Smith, 2003; Owen et al., 2005; Barbey et al., 2011). Studies of human brain lesion patients with damage to orbitofrontal cortex have shown specific behavioral outcome deficits on components central to working memory (Barbey et al., 2011). Orbitofrontal damage was associated with deficits on working memory tasks involving coordination of maintenance, manipulation, and monitoring processes (e.g., N-Back task). However, this association was not seen on neuropsychological tests of working memory maintenance (digit/spatial span forward) or manipulation (digit/spatial span backward and letter-number sequencing; Barbey et al., 2011).

## Limitations

Although not significantly different on age, the low performers tended to be older than high performers. If the sample size increased, it is possible this could impact the overall results as structural brain changes increase in older age. Even still, age was used in our models as a covariate to account for any numerical differences in age between groups. It is also possible that the clinical assessment of MCI by the study neuropsychologist did not capture participants in the earliest stages of MCI. This possibility is supported by the range of MoCA scores in this study, although these ranges were not significantly different between groups. Nonetheless, our findings may by biased by an unknown number of participants in either group that were in the earliest stages of MCI and thus evidencing early neurodegenerative tissue loss. The N-Back may also exhibit limitations inherent to the task regarding its use for the study of lateralized differences in structure-function relationships. As the functional foci of activation for N-Back changes with age and development, this tool may not be ideal for full identification of all frontal related working memory related neural correlates.

## Conclusion

Normal physiological processes of aging are associated with neuronal circuitry changes, which may result in impaired cognition and behavior in some older individuals. Individuals that show poorer cognitive performance tend to show impairments of executive functions first (e.g., working memory, planning, and goal directed behavior), thus it has been postulated that neurons and circuits of the prefrontal cortex may be particularly vulnerable during normal aging in humans and non-human primates (Dickstein et al., 2013). During the aging process, there is evidence that neurons undergo morphological changes such as reduced complexity of dendritic arborization and dendritic length, as well as decreases in spine numbers. As spines are the major sites for excitatory synapses, changes in spine numbers could reflect a change in synaptic densities (Dickstein et al., 2007). These morphological changes may underlie surface area reductions, as neuron numbers remain relatively stable in older aged individuals lacking neurodegenerative diseases. As dendrites are pivotal in forming and maintaining neural networks, regulating synaptic plasticity, and integrating electrical inputs (Dickstein et al., 2007), it is perhaps not surprising that a potential marker of age-related change in dendritic morphology correlates with poorer performance on behavioral tasks.

There is great variability in cytoarchitectonic features of the cortex between individuals (Kringelbach and Rolls, 2004). The difficulty of deciphering the functional role of any brain region lies in the complexities of connections between and within brain structures, which may lend a single structure the ability to activate for a multitude of tasks. The N-Back task used in this study requires considerable vigilance and working memory processes to accurately detect target letters in the correct 2-Back pattern, a task that is quite challenging.

The right inferior frontal gyrus, an area implicated in cognitive inhibition and working memory, demonstrated significant reduction in surface area in older adults with lower working memory performance. The SFG, a crucial substrate of working memory processes, also exhibited a reduction in structural integrity. Finally, the MOF, a region shown to be necessary in coordination of working memory maintenance, manipulation, and monitoring processes also exhibited significantly reduced cortical surface area in low working memory groups. Taken together, these regions appear to play an important role in age-related working memory decline. The structural integrity of these three regions may also play an important role relative to compensatory processes previously found in functional MRI studies of N-Back performance. For example, deficits in these right frontal regions may interfere with compensatory engagement of left frontal structures found to activate in older adults able to maintain a high level of working memory performance. Future research investigating differences in both functional and structural connectivity between right and left frontal regions in high versus low working memory performers will be important for further evaluating this theory. In addition, these three frontal areas may prove to be important therapeutic targets for brain stimulation or other methods capable of upregulating cerebral metabolism and function in brain regions showing decline.

## Ethics Statement

The Institutional Review Board (IRB) at the University of Florida approved this study. Prior to any study procedures, all participants provided written informed consent. The study protocol was carried out in accordance with the Declaration of Helsinki, and the University of Florida Institutional Review Board approved all procedures in this study. All study participants were healthy older adults.

## Author Contributions

NN, AO, RC, EP, VB, and AW contributed text to the manuscript. AO, NN, and AW performed data analysis. All authors provided edits and approved the final version of the manuscript.

## Conflict of Interest Statement

The authors declare that the research was conducted in the absence of any commercial or financial relationships that could be construed as a potential conflict of interest.

## Footnotes

1 https://www.r-project.org/
